# Mechanistic insights into the PAI-1 inhibitor PAItrap3: enhancing lipid metabolism in adipose tissue of diabetic db/db mice

**DOI:** 10.3389/fphar.2025.1596655

**Published:** 2025-06-12

**Authors:** Linxi Wang, Zhouyangyang Zhang, Menghua Lin, Liqin Qi, Libin Liu, Zhuo Chen, Shuzhi Tang, Lijing Wang

**Affiliations:** ^1^ Department of Endocrinology and Metabolism, Fujian Institute of Endocrinology, Fujian Medical University Union Hospital, Fuzhou, China; ^2^ State Key Laboratory of Structural Chemistry, Fujian Institute of Research on the Structure of Matter, Chinese Academy of Sciences, Fujian College, University of Chinese Academy of Sciences, Fuzhou, China; ^3^ Fujian Provincial Key Laboratory of Ecology Toxicological Effects and Control for Emerging Contaminants, Key Laboratory of Ecological Environment and Information Atlas, Fujian Provincial University, College of Environmental and Biological Engineering, Putian University, Putian, Fujian, China

**Keywords:** PAI-1 inhibitor, lipid metabolism, autophagy, energy metabolism, diabetes mellitus

## Abstract

**Objective:**

This study aimed to investigate the effects of PAItrap3, a novel PAI-1 inhibitor, on lipid metabolism, and autophagy pathways in diabetic mice.

**Methods:**

db/db diabetic mice were administered PAItrap3 (5.7 mg/kg/day, IV) for 21 consecutive days, and its impact on metabolic, gene expression, and lipidomic profiles was assessed. Western blot analysis was performed to examine lipid metabolism-related proteins in white adipose tissue (FASN, HSL, CPT1A, ACADM) and autophagy markers (LC3B, P62, Parkin, PGC1α, PPARGC1B). Additionally, RNA-seq and targeted lipidomics were employed to analyze gene expression and lipid metabolic alterations.

**Results:**

PAItrap3 significantly reduced blood glucose and glycated hemoglobin levels while improving insulin sensitivity. In lipid metabolism, FASN and HSL levels were upregulated, whereas CPT1A and ACADM levels were downregulated in the DMP group. Regarding the autophagy pathway, PPARGC1B, LC3B, and PGC1α expression levels were increased, while P62 and Parkin levels were decreased. Lipidomics analysis revealed that triglycerides (TG) and diacylglycerols (DG) were generally downregulated, with TG (18:2/18:2/18:2) (0.96 [0.8491, 1]), LPI (18:0) (0.96 [0.8491, 1]), and MLCL (14:3/20:4/22:6) (0.96 [0.8491, 1]) identified as key metabolites.

**Conclusion:**

This study finds that PAItrap3 modulates lipid metabolism, energy homeostasis, and autophagy pathways, thereby improving metabolic dysfunction in diabetic mice. These findings highlight its potential therapeutic value for treating diabetes-associated lipid metabolic disorders.

## 1 Introduction

Diabetes mellitus is not only a disorder of glucose metabolism but is also accompanied by significant lipid metabolism dysfunction. In patients with type 2 diabetes mellitus (T2DM), dyslipidemia is a common metabolic abnormality characterized by elevated triglycerides (TG), increased low-density lipoprotein cholesterol (LDL-C), and decreased high-density lipoprotein cholesterol (HDL-C). These metabolic imbalances collectively contribute to insulin resistance, non-alcoholic fatty liver disease (NAFLD), atherosclerosis, and cardiovascular diseases (Eckel et al., 2005). Adipose tissue, a key energy storage and endocrine organ, is tightly regulated by insulin signaling. Under conditions of insulin resistance, lipolysis is accelerated, leading to elevated levels of free fatty acids (FFAs), which further exacerbate insulin resistance (Boden, 2011). Moreover, lipid metabolism dysfunction not only impairs glucose utilization in peripheral tissues but also induces β-cell apoptosis via lipotoxicity, creating a vicious metabolic cycle (Kusminski et al., 2009). Therefore, targeting lipid metabolism regulation has become a crucial strategy for diabetes management and has been incorporated into the latest clinical guidelines (American Diabetes Association Professional Practice and C 8, 2024).

In the pathophysiology of diabetes, inflammatory responses in adipose tissue, lipid droplet metabolism dysregulation, and impaired autophagy are considered key mechanisms. For instance, decreased adiponectin levels, leptin resistance, and elevated pro-inflammatory cytokines such as TNF-α and IL-6 all contribute to insulin resistance (Castela et al., 2023). Additionally, autophagy dysfunction, particularly imbalanced expression of key autophagy-related proteins such as LC3B, Atg5, and Atg7, plays a crucial role in lipid storage and breakdown (Singh et al., 2009). Thus, interventions targeting lipid metabolism disorders hold significant clinical and therapeutic potential for improving metabolic health in diabetic patients.

Plasminogen Activator Inhibitor-1 (PAI-1) is a key adipose tissue-derived factor that not only plays a critical role in thrombosis formation but is also strongly associated with obesity, insulin resistance, and lipid metabolism disorders (Asish and GDouglas, 2011). Studies have demonstrated that PAI-1 expression is significantly elevated in obese individuals and is positively correlated with visceral fat accumulation and reduced insulin sensitivity (Flavia Campos et al., 2011; Fang et al., 2024). Furthermore, PAI-1 can inhibit angiogenesis and activate the NF-κB inflammatory signaling pathway, thereby exacerbating metabolic dysregulation (De Craen and Declerck, 2012). PAI-1 inhibitors have gained increasing attention for their therapeutic potential across a range of metabolic and inflammatory conditions. In cardiovascular diseases, for example, these inhibitors have been shown to reduce thrombus formation, alleviate atherosclerosis, and ultimately lower the risk of adverse cardiovascular events (Taeye et al., 2005; Sillen, 2020). In the context of nonalcoholic fatty liver disease (NAFLD), PAI-1 overexpression is known to contribute to hepatic steatosis by promoting lipid accumulation and disrupting lipid homeostasis. Notably, recent studies demonstrate that inhibiting PAI-1 can reverse these effects by restoring hepatic lipid metabolism and mitigating excessive fat deposition (Levine et al., 2021a). Furthermore, inhibiting PAI-1 can restore the expression of mitochondrial biogenesis-related genes in HepG2 liver cells, thereby improving mitochondrial function and enhancing hepatic energy metabolism (Lee et al., 2017).

Notably, recent studies have demonstrated that PAI-1 inhibitors not only ameliorate insulin resistance but may also exert direct hypoglycemic effects, positioning them as potential therapeutic agents for diabetes treatment. Furthermore, PAI-1 inhibitors have been proposed as novel antithrombotic agents that could help reduce vascular complications in diabetic patients, offering new therapeutic avenues for managing diabetes-associated cardiovascular risks (Altalhi et al., 2021). Additionally, certain PAI-1 inhibitors have been shown to enhance insulin secretion, promote pancreatic β-cell survival, and indirectly improve insulin sensitivity by suppressing pro-inflammatory cytokines (Tang et al., 2020).

Although previous studies have demonstrated the critical role of PAI-1 in glucose metabolism dysregulation and diabetes progression, the potential of PAI-1 inhibitors in regulating lipid metabolism in diabetes remains largely unexplored. Therefore, this study aims to establish an db/db diabetic mouse model and administer a novel PAI-1 inhibitor (PAItrap3) to investigate its regulatory effects beyond glycemic control, particularly on energy metabolism in adipose tissue and autophagy-related pathways. This study represents the first systematic evaluation of PAItrap3 in modulating lipid metabolism and autophagy in diabetes and aims to uncover its underlying molecular mechanisms, thereby providing new scientific insights for the precision treatment of diabetes and related metabolic disorders.

## 2 Methods

### 2.1 Materials

Six-week-old db/m and db/db mice were obtained from Gene and Peace Biotech Co., Ltd., (Jiangsu, China). Blood glucose levels were measured using a glucometer and test strips from Abbott Diabetes Care, UK. ELISA kits for mouse insulin (INS), glycated hemoglobin (GHb), nuclear factor κB (NF-κB), tumor necrosis factor-α (TNF-α), interleukin-6 (IL-6), triglycerides (TG), total cholesterol (TC), and free fatty acids (FFAs) were purchased from Quanzhou Ruixin Biotechnology Co., Ltd., (Quanzhou, China). Antibodies for Parkin and LC3B were obtained from Cell Signaling Technology (catalog numbers: 2132S and 83506S, MA, United States); P62 antibody was from Abcam (catalog number: ab56416, Cambridge, MA, United States); PPARGC1B, ACADM, and CPT1A antibodies were from Proteintech (catalog numbers: 22378-1-AP, 55210-1-AP, and 15184-1-AP, Wuhan, China); FASN, PGC1α, and HSL antibodies were from ABclonal (catalog numbers: A19050, A12348, and A24689, Wuhan, China); and β-actin antibody was from Affinity Biosciences (catalog number: T0022, Jiangsu, China). HRP-conjugated anti-rabbit IgG and anti-mouse IgG secondary antibodies were purchased from Boster Biological Technology (Wuhan, China). qRT-PCR kits, reverse transcription kits, and RNA extraction kits were obtained from Vazyme (Nanjing, China). The preparation of the PAI-1 inhibitor PAItrap3 is detailed in the referenced literature (Tang et al., 2020).

### 2.2 Animals model establishment and grouping design

**FIGURE 1:**
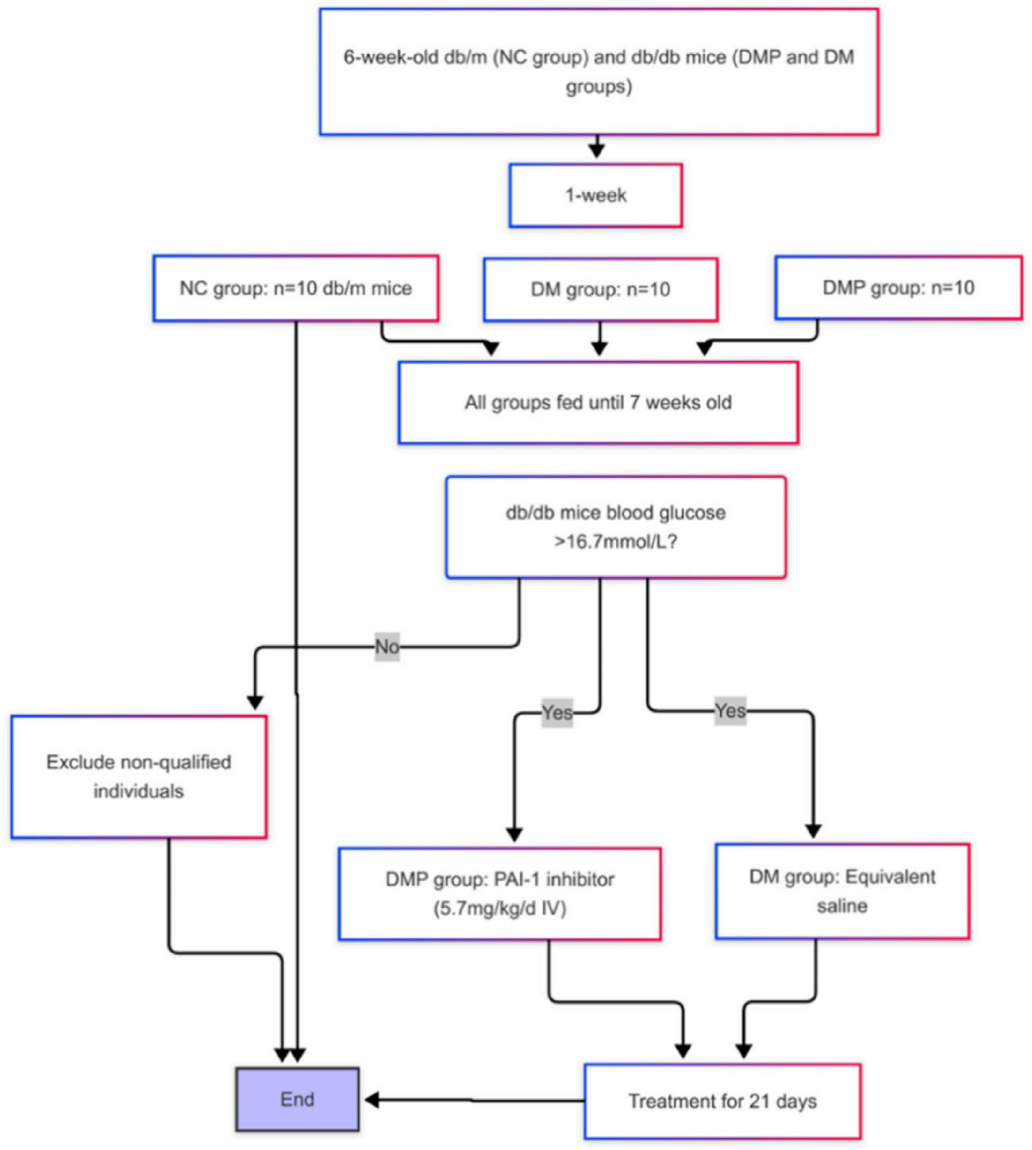
Animals model establishment and grouping design.

Db/m and db/db mice were randomly assigned into three groups: the normal control group (NC group), the diabetes group (DM group), and the diabetes with treatment group (DMP group). After 1 week of adaptive feeding, 20 male db/db mice were randomly divided into DM and DMP groups (10 mice per group), while 10 db/m mice were assigned to the NC group. At approximately 7 weeks of age, random blood glucose levels were measured. Once the random blood glucose in db/db mice exceeded 16.7 mmol/L (Wang et al., 2023), administration of the PAI-1 inhibitor was initiated (Lin et al., 2025). The DMP group received PAI-1 inhibitor at a dose of 5.7 mg/kg/day via intravenous (IV) injection for 21 consecutive days, while the DM group was injected with an equivalent volume of normal saline (Figure 1). The mice were euthanized after intraperitoneal injection of 2% pentobarbital sodium. The peritoneal fat pad masses were rapidly excised, weighed, and flushed with ice-cold saline. A portion of the tissue was fixed in 10% neutral formalin solution for histological analysis, while the remaining portion was immediately stored at −80°C for subsequent analysis. All animal protocols were approved by the Fujian Medical University Institutional Animal Care and Use Committee (FJMU IACUC 2021-0029).

### 2.3 HE staining of adipose tissue

Fresh adipose tissue was fixed in 4% paraformaldehyde at 4°C for 24 h, washed three times with PBS (10 min per wash), then dehydrated through a graded ethanol series (70%, 85%, 95%, 100%), cleared in xylene, and embedded in paraffin. Sections (5 μm thickness) were prepared, baked at 60°C for 2 h, dewaxed in xylene (2 × 10 min), and rehydrated through a graded ethanol series. Nuclei were stained with Harris hematoxylin for 5 min, followed by bluing in running water for 10 min. Cytoplasm was counterstained with 0.5% eosin for 1 min, followed by dehydration through a graded ethanol series, clearing in xylene, and mounting with neutral resin. Note: Complete dewaxing is critical to avoid lipid droplet retention; eosin staining duration may be adjusted based on tissue thickness.

### 2.4 Oil red O staining of adipose tissue

Frozen sections (5 μm) of adipose tissues were first washed in phosphate-buffered saline (PBS) and subsequently fixed with paraformaldehyde (4%) for 30 min. The samples were incubated in 60% (vol/vol) isopropyl alcohol for 3 min, followed by staining with freshly prepared 60% oil red O (100% solution: 0.5 g of oil red O dissolved in 100 mL of isopropylene) for 30 min and 60% (vol/vol) isopropyl alcohol for 1 min and a wash with PBS. The samples were counterstained with hematoxylin. The slides were visualized using an Olympus microscope (Olympus, Tokyo, Japan). Lipid droplet (LD)number and LD size were quantified using ImageJ software following standard protocols (for oil red O staining, at least 100 cells of each group were analyzed).

### 2.5 Lipid targeted metabolomics

Samples were mixed with 280 μL methanol (2:5, v/v) solution and 400 μL MTBE. After grinding (6 min, −20°C, 50 Hz), ultrasonication (30 min, 5°C, 40 kHz), and centrifugation (13,000 g, 15 min, 4°C), 350 μL lipid extracts in the upper phase were transferred to new tubes and evaporated to dryness. The samples were reconstituted in 100 μL loading solution of isopropanol (1:1, v/v) by brief sonication in a 5°C water bath. The extracted lipids were centrifuged (13,000 g, 15 min, 4°C), and the supernatant was transferred to a sample vial for UHPLC-MS/MS analysis. Chromatographic separation of the lipids was performed using a Thermo UHPLC-Q Exactive HF-X Vanquish Horizon system equipped with an Accucore C30 column (100 mm × 2.1 mm i. d., 2.6 μm) thermostated at 40°C. Separation of the lipids was achieved at a 0.4 mL/min flow rate with a mobile phase gradient consisting of 10 mM ammonium acetate in acetonitrile (1:1, v/v) containing 0.1% formic acid, and 2 mM ammonium acetate in acetonitrile:isopropanol(10:88:2, v/v/v) containing 0.1% formic acid. The total chromatographic separation time was 20 min. The mass spectrometric data were collected using a Thermo UHPLC-Q-Exactive HF-X Benchtop Orbitrap Mass Spectrometer, and data acquisition was performed using Data Dependent Acquisition (DDA) mode. The detection was carried out over a mass range of 200–2000 m/z (Qingsong et al., 2023).

### 2.6 Data capture and preprocessing

Raw data were processed using Progenesis QI (Waters Corporation, Milford, United States), and peaks were then matched to the metabolic public databases HMDB, Metlin, and the database built by Majorbio company for accurate qualitative and relative quantitative results. Data were analyzed through the free online platform of the Majorbio Cloud Platform. The specific metabolomics methods are provided in the Supplementary Material (Chang et al., 2024).

### 2.7 RNA-seq

Total RNA was extracted from three biological replicates of mise treated as indicated. RNA-sequencing (RNA-seq) analysis was performed at Majorbio Bio-Pharm Technology Co., Ltd. (Shanghai, China). Differential gene expression among the samples was established using DESeq2 (P adjust< 0.05, |fold change| > 1.5). Functional enrichment of GO (Gene Ontology) terms and KEEG (Kyoto Encyclopedia of Genes and Genomes) pathways was then performed (Wu S. et al., 2024).

### 2.8 ELISA

After weighing, adipose tissue was mixed with pre-chilled PBS and protease inhibitors at a volume ratio of 1:9:1, and thoroughly homogenized on ice. The homogenate was then centrifuged at 5,000×*g* for 5–10 min, and the supernatant was collected for further analysis. The supernatants were screened using a commercial ELISA kit (Quanzhou Ruixin Biotechnology Co., Ltd., China) according to the manufacturer’s instructions and the absorbance was finally measured at 450 nm using an EL×800 microplate reader.

### 2.9 Real-time quantitative polymerase chain reaction (qRT-PCR)

The target gene sequences for, ACC1, Perilipin, Slc27a5, PAI-1, as well as the housekeeping gene Gapdh, were retrieved from GenBank. Primers were designed using Primer 3.0 software following standard primer design principles and verified for specificity using NCBI BLAST, then synthesized by a commercial provider (sequences shown in Table 1). The qRT-PCR reaction was performed under the following conditions: initial denaturation at 95°C for 1 min, followed by 40 cycles of denaturation at 94°C for 20 s, annealing at 60°C for 20 s, and extension at 72°C for 30 s, with a final 95°C for 15 s, 60°C for 1 min, 95°C for 15 s, and 60°C for 15 s. Total RNA was extracted, and qRT-PCR was conducted according to the reagent kit instructions, using Gapdh as an internal control. The relative mRNA expression levels of target genes were calculated using the ΔCt method, where two technical replicates per sample were performed to obtain Ct values. The mean Ct value for each target gene was subtracted from the corresponding Gapdh Ct value (ΔCt = Ct_target gene - Ct_Gapdh) to determine the relative gene expression.

**TABLE 1:** The sequences of primers.

Gene	5′–3′
β-actin	F: AAC​AGT​CCG​CCT​AGA​AGC​AC
	R: CGT​TGA​CAT​CCG​TAA​AGA​CC
ACC1	F: ATG​TTG​AGA​CGC​TGG​TTT​GTA​G
	R: TCT​TCC​TCT​GTC​AGT​TGC​TTC​T
Perilipin	F: ATG​CCC​TGA​AGG​GTG​TTA​CG
	R: TGT​CTC​GGA​ATT​CGC​TCT​CG
Slc27a5	F: TTG​GAT​TCC​TTG​GCT​GCT​TAC
	R: CGC​ACT​GTA​TGT​ATC​TTG​TCT​TCT
PAI-1	F:ATCGAGGTAAACGAGAGCGG
	R:TTGTCTCTGTCGGGTTGTGC

### 2.10 Western blot

Adipose tissues from mice were lysed using RIPA lysis buffer containing 1% PMSF and 1% protease inhibitor cocktail (both from Sigma-Aldrich), and total protein concentrations were determined using the bicinchoninic acid (BCA) assay. Protein samples were blocked in 5% nonfat milk in Tris-buffered saline (TBS) for 2 h, followed by incubation with primary antibodies against PGC1α, p62, Parkin, LC-3B, PPARGC1B, ACADM, CPT1A, HSL (1:1,000), FASN (1:500), and β-actin (1:2,000) at 4°C overnight. The next day, HRP-conjugated secondary antibodies (1:2,000) were applied for 1 h at room temperature, and protein bands were detected using electrochemiluminescence (ECL) reagents. Imaging was performed using a BIO-RAD gel imaging system (United States), and experiments were repeated three times with consistent results. Protein band intensities were quantified using Quantity One analysis software (BIO-RAD, United States).

### 2.11 Statistical analysis

Data are presented as mean (SD). Comparisons between groups were performed using a two-tailed t-test analysis, one-way ANOVA, or two-way ANOVA with repeated measures. P < 0.05 was considered statistically significant.

## 3 Result

### 3.1 Effects of PAItrap3 on metabolic and inflammatory markers in diabetic mice

Previous studies demonstrated that PAItrap3 treatment reduced blood glucose, TNF-α (Tang et al., 2020), and lipid levels (Lin et al., 2025) in diabetic mice, along with a significant decrease in lipid levels. To validate these findings, two additional diabetic mice were analyzed, yielding consistent results. In this study, compared with the normal control group (NC group), diabetic mice (DM group) exhibited significantly higher body weight, blood glucose, glycated hemoglobin (GHb), and inflammatory markers (NF-kB, TNF-α, and IL-6), whereas insulin levels were markedly reduced (P < 0.01). Body weight in the DMP group showed no statistically significant difference compared to the DM group (P > 0.05). While blood glucose and glycated hemoglobin levels significantly decreased (P < 0.01), and insulin levels increased (P < 0.05), suggesting that PAItrap3 may restore pancreatic function and improve glycemic control. Furthermore, NF-kB (P < 0.01), TNF-α, and IL-6 (P < 0.05)were significantly reduced, indicating that PAItrap3 may alleviate metabolic dysfunction in diabetes by suppressing inflammatory pathways (Table 2).

**TABLE 2:** Effects of PAItrap3 on metabolic and inflammatory markers in diabetic mice.

characteristic	NC group	DM group	DMP group
Weight (g)	26.08 ± 2.24	41.32 ± 1.40^**^	44.96 ± 2.70
Glucose (mmol/L)	9.16 ± 1.15	27.22 ± 1.30^**^	17.18 ± 3.99^###^
Insulin (mIU/L)	5.20 ± 0.78	2.74 ± 1.00^**^	4.50 ± 0.82^#^
GHb (ng/mL)	41.92 ± 7.24	56.55 ± 5.84^*^	44.85 ± 7.37^#^
NF-kB (pg/mL)	91.00 ± 6.18	145.58 ± 24.22^**^	103.33 ± 16.96^##^
TNF-α (pg/mL)	62.37 ± 4.97	104.44 ± 12.45^**^	80.92 ± 12.66^#^
IL-6 (pg/mL)	11.52 ± 1.12	19.38 ± 1.73^**^	15.80 ± 1.93^#^
TG (mmol/L)	0.40 ± 0.05	0.75 ± 0.00	0.43 ± 0.01
TC (mmol/L)	1.40 ± 0.17	2.00 ± 0.04	1.40 ± 0.12
FFA (μmol/mL)	17.75 ± 0.82	40.56 ± 1.88	33.58 ± 1.67

n = 5; *P < 0.05, **P < 0.01 vs. NC, group; #P < 0.05, ##P < 0.01 vs. DM, group.

NC: Control group, DM: Diabetes mellitus group, DMP: PAItrap3 treatment group.

### 3.2 Adipose tissue HE staining, Oil red O Staining, and PAI-1 level

**FIGURE 2:**
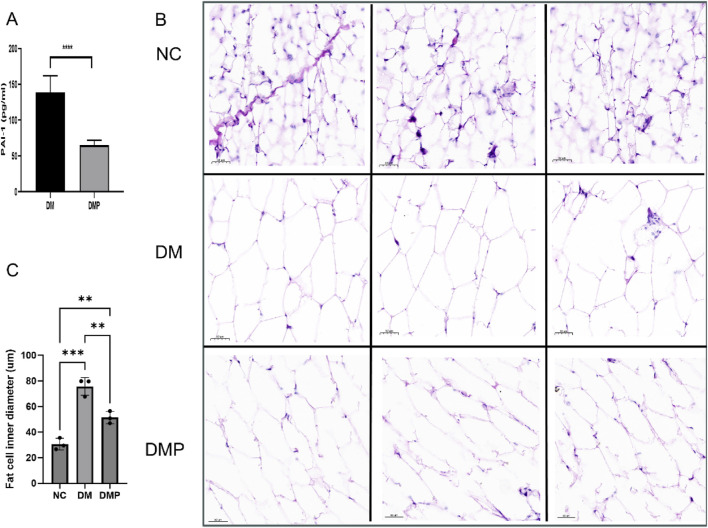
Adipose Tissue HE staining and PAI-1 level. **(A)** PAI-1 level measured using an ELISA kit (n = 6). **(B)** HE staining of adipose tissue in different groups. **(C)** Bar graph showing adipocyte diameter fraction. NC: Control group, DM: Diabetes mellitus group, DMP: PAItrap3 treatment group. **P < 0.001, ***P < 0.0001; n = 3.

**FIGURE 3:**
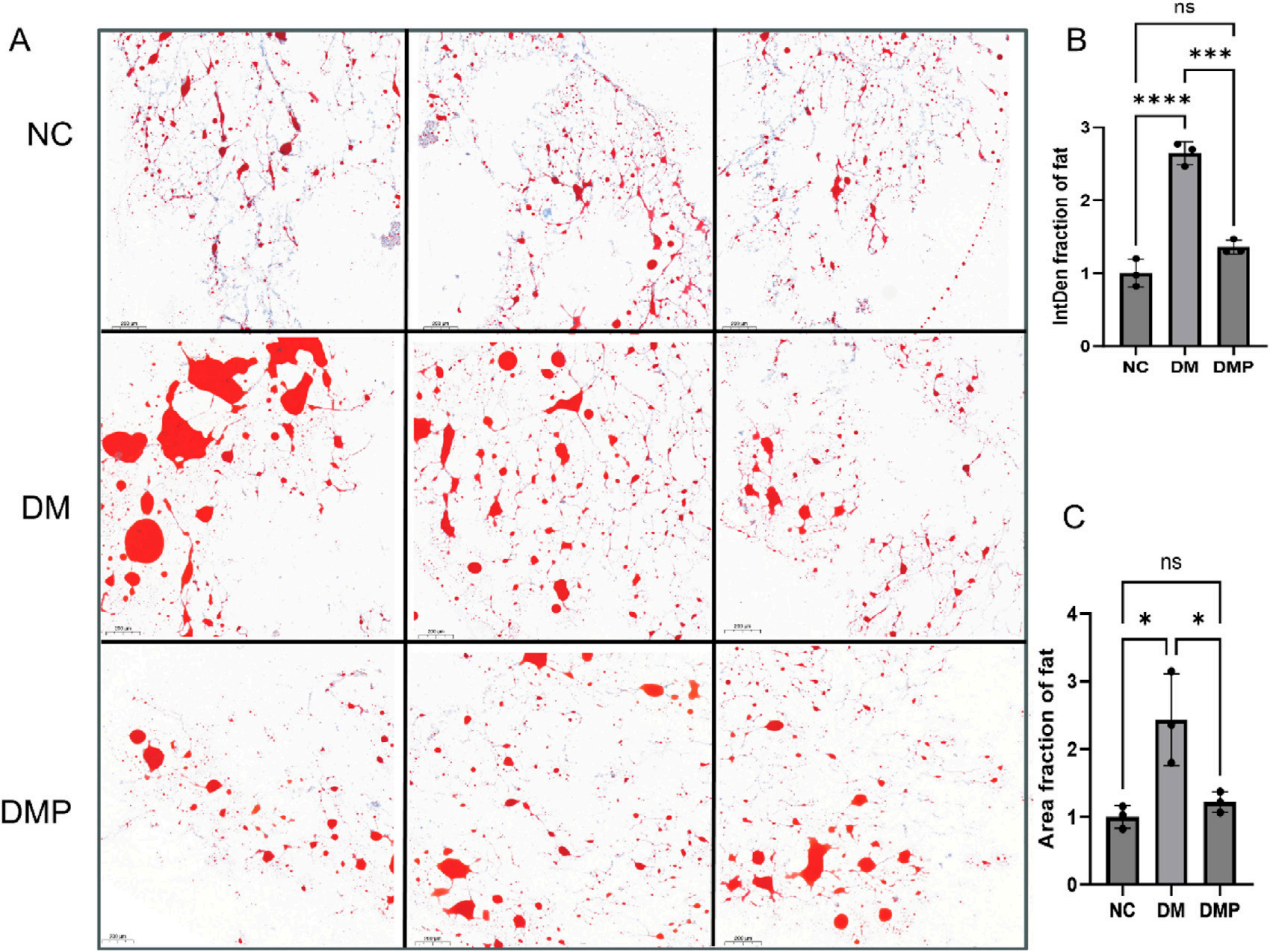
Adipose Tissue Oil Red O staining. **(A)** Oil Red O staining of adipose tissue in different groups, with red areas indicating lipid deposition. **(B)** Bar graph showing integrated density (IntD) fraction of adipocytes. **(C)** Bar graph showing area fraction of fat. NC: Control group, DM: Diabetes mellitus group, DMP: PAItrap3 treatment group. *P < 0.05, **P < 0.001, ***P < 0.0001; n = 3.

We measured the concentration of PAI-1 in adipose tissue. Following PAItrap3 treatment, PAI-1 levels in the DMP group were significantly reduced compared to the DM group (139.00 ± 20.95 pg/mL vs. 65.17 ± 6.10 pg/mL, p < 0.01), indicating effective inhibition of PAI-1 in adipose tissue. (Figure 2A). HE staining revealed that adipocyte diameter in the DM group was significantly larger than that in the NC group (Figures 2B,C). Compared with the DM group, adipocyte diameter in the DMP group was significantly reduced. Oil Red O staining (Figure 3) further demonstrated that the number and size of orange-red lipid droplets in adipose tissue were significantly increased in the DM group compared to the NC group. However, in the DMP group, both the number and volume of lipid droplets were significantly reduced compared to the DM group, though still higher than those in the NC group. These findings indicate that PAItrap3 treatment reduces adipocyte size and lipid droplet accumulation.

### 3.3 RNA-seq of db/db mice adipose tissue

**FIGURE 4:**
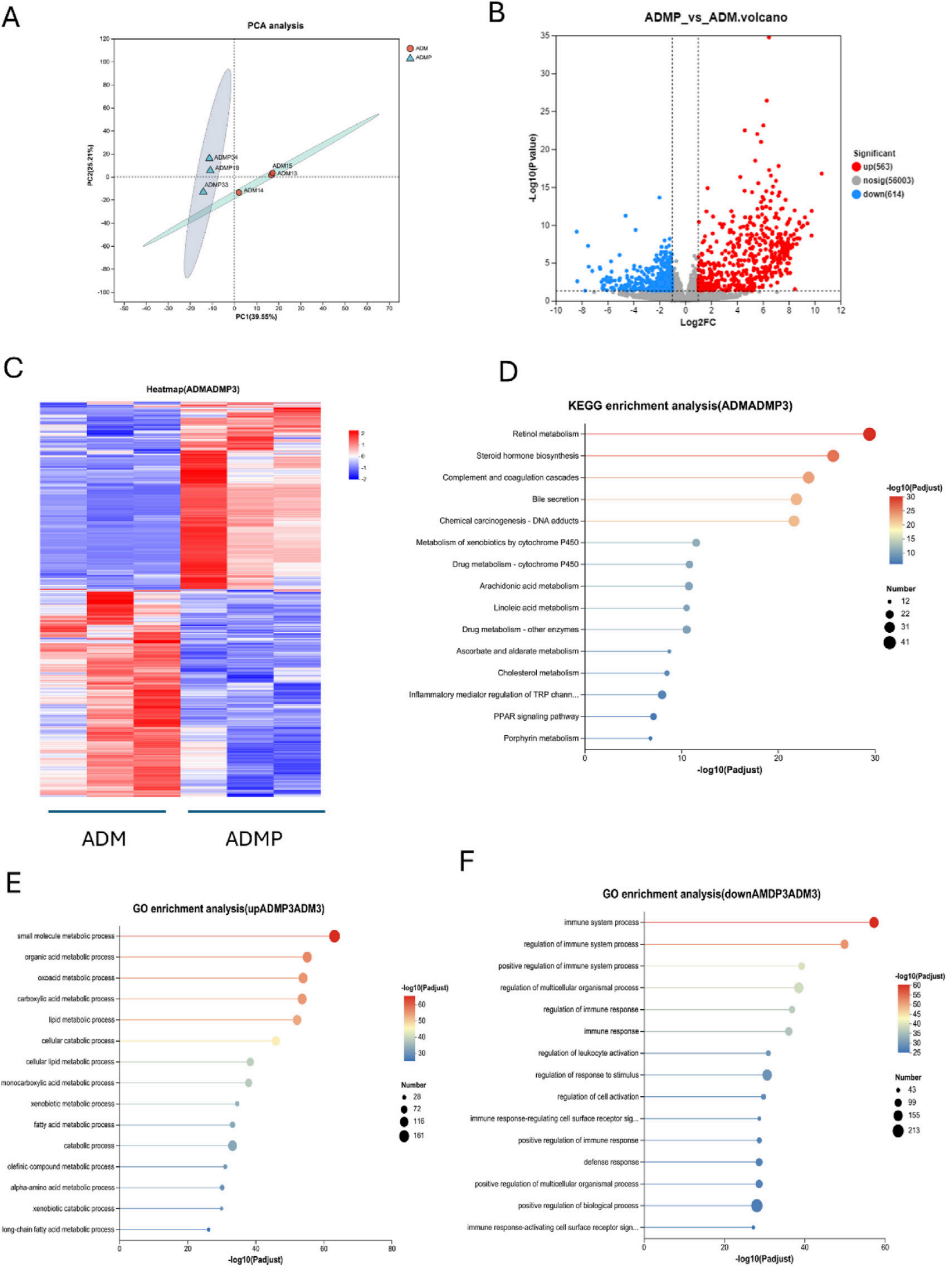
RNA-seq Analysis Results. **(A)** Principal Component Analysis (PCA), **(B)** Volcano Plot, The x-axis represents the fold change in gene expression between the two groups, denoted as log2FC, while the y-axis represents the statistical test value of the difference in gene expression, denoted as −log10 (p_value). Red points represent metabolites that are significantly upregulated compared to the control, blue points represent significantly downregulated metabolites, and gray points indicate metabolites with no significant difference. **(C)** Metabolite Clustering Heatmap, Each column represents a sample, and each row represents a metabolite. The colors in the figure represent the relative expression levels of metabolites in each sample group. **(D)** KEGG Pathway Enrichment Analysis of Differential Genes. **(E,F)** GO Pathway Enrichment Analysis of Differential Genes. ADM: Diabetes mellitus group, ADMP: PAItrap3 treatment group. *P < 0.05, **P < 0.01, ***P < 0.001. n = 3.

To further investigate the molecular mechanisms underlying PAItrap3-mediated metabolic regulation in adipose tissue, we performed RNA-seq analysis on adipose tissues from DM (ADM) and DMP (ADMP) groups. PCA analysis revealed that samples within each group clustered closely, indicating high intra-group similarity, whereas the ADM and ADMP groups were clearly separated, suggesting significant inter-group differences in gene expression (Figure 4A). Volcano plot analysis further identified 563 upregulated and 614 downregulated genes in the ADMP group compared to the ADM group (Figure 4B). Among these, genes closely related to lipid metabolism that were significantly upregulated in the ADMP group included Slc27a5, Apoa1, Fabp1, Baat, Apoc3, Fabp2, Cyp7a1, Cyp8b1, Acnat2, Cideb, Acox2, Acot3, Angptl3, and Mttp, whereas Soat1 and Acot11 were downregulated. A complete list of differentially expressed genes can be found in the Supplementary Table S1. Hierarchical clustering heatmaps revealed distinct gene expression patterns before and after treatment (Figure 4C). KEGG pathway enrichment analysis, based on p-value ranking, indicated significant enrichment in retinol metabolism, steroid hormone biosynthesis, complement and coagulation cascades, and bile secretion. Additionally, pathways closely related to lipid metabolism, such as cholesterol metabolism, PPAR signaling pathway, primary bile acid biosynthesis, and fat digestion and absorption, were also significantly enriched (Figure 4D; Supplementary Table S2). GO enrichment analysis was further utilized to associate differentially expressed genes with biological processes and disease pathways. The downregulated genes in the ADMP group were primarily associated with immune system processes, regulation of immune system processes, immune response, and regulation of leukocyte activation (Figure 4E). In contrast, the upregulated genes in the ADMP group were significantly enriched in small molecule metabolic processes, organic acid metabolism, oxoacid metabolism, carboxylic acid metabolism, and lipid metabolic processes (Figure 4F). These findings suggest that PAItrap3 may regulate metabolic homeostasis by modulating immune responses and lipid metabolism in adipose tissue.

### 3.4 qRT-PCR

RNA-seq analysis revealed that PAItrap3 treatment was closely associated with lipid metabolism-related pathways in adipose tissue. To validate the RNA-seq findings, we examined the expression of Slc27a5, which was consistently upregulated in the DMP group, confirming the reliability of the sequencing results. Additionally, PAI-1 expression was reduced in the DMP group, aligning with our previous experimental findings. Furthermore, based on KEGG-enriched lipid metabolism-related genes identified in RNA-seq, we conducted additional screening. The results showed that Acc1 and Perilipin expression levels were decreased, but the differences were not statistically significant (Table 3).

**TABLE 3:** Gene expression profile in adipose tissue.

Gene	DM	DMP
Acc1	1.01 ± 0.18	1.1 9 ± 0.58
Slc27a5	1.77 ± 1.93	4.08 ± 2.47
PAI-1	1.06 ± 0.34	0.92 ± 0.10
Perilipin	1.36 ± 1.11	1.25 ± 0.77

DM: diabetes mellitus group, DMP: PAItrap3 treatment group. n = 3.

### 3.5 Lipid-targeted metabolomics

**FIGURE 5:**
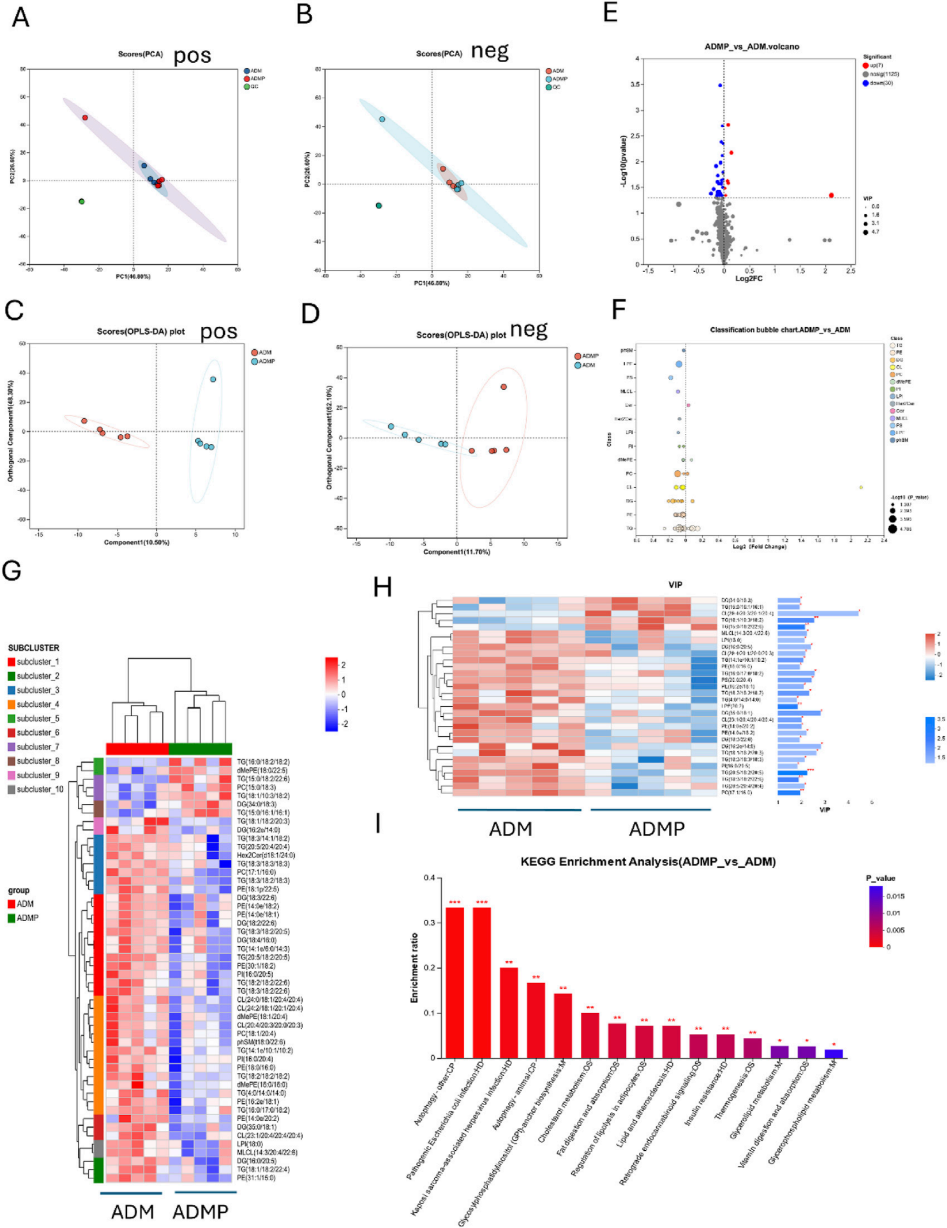
Lipid-Targeted Metabolomics Analysis Results. **(A,B)** Principal Component Analysis (PCA), **(C,D)** (OPLS-DA), **(E)** Volcano Plot, The x-axis represents the fold change in metabolite expression between the two groups, denoted as log2FC, while the y-axis represents the statistical test value of the difference in metabolite expression, denoted as −log10(p_value). Red points represent metabolites that are significantly upregulated compared to the control, blue points represent significantly downregulated metabolites, and gray points indicate metabolites with no significant difference. **(F)** Lipid Classification Analysis, The Y-axis represents different lipid subclasses, and the X-axis represents the sum of the contents of different lipid metabolites within the same lipid class. Bars of different colors represent different groups. **(G)** Metabolite Clustering Heatmap, Each column represents a sample, and each row represents a metabolite. The colors in the figure represent the relative expression levels of metabolites in each sample group. **(H)** VIP Score Plot of Metabolites, On the left is the metabolite clustering dendrogram; closer branches indicate that the expression patterns of all metabolites within the samples are more similar. Each column represents a sample, with sample names displayed at the bottom. Each row represents a metabolite, with colors indicating the relative expression levels of that metabolite within the sample group. The correspondence between color gradient and value size can be seen in the gradient color bar. The asterisks on the right denote statistical significance: * indicates P < 0.05, ** indicates P < 0.01, *** indicates P < 0.001. **(I)** KEGG Pathway Enrichment Analysis of Differential Metabolites. ADM: Diabetes mellitus group, ADMP: PAItrap3 treatment group. n = 5.

Building upon prior RNA-seq and RT-PCR results, which indicated significant involvement of lipid metabolism pathways, we performed lipid-targeted metabolomics analysis on adipose tissues from diabetic mice before and after PAItrap3 treatment. Principal component analysis (PCA) demonstrated that all QC samples clustered tightly on the score plot, indicating good data reproducibility and consistency (Figures 5A,B). Orthogonal partial least squares-discriminant analysis (OPLS-DA) permutation test revealed that lipid metabolic patterns were completely separated before and after PAItrap3 intervention, with pos (R^2^X (cum): 0.588, Q^2^ (cum): 0.651) and neg (R^2^X (cum): 0.638, Q^2^ (cum): 0.699) (Figures 5C,D). Volcano plot analysis identified 30 significantly downregulated and 7 significantly upregulated lipid metabolites in the ADMP group compared to the ADM group (Figure 5E). Most triglycerides (TGs) were downregulated, except for TG(15:0/16:1/16:1), TG (15:0/18:2/22:6), TG (18:1/10:3/18:2), and TG (16:0/18:2/18:2), which were upregulated. Among diacylglycerols (DGs), only DG (34:0/18:3) was upregulated. Most cardiolipins (CLs) were downregulated, except for CL (20:4/20:3/20:1/20:4), which was upregulated. Additionally, all phosphatidylethanolamines (PEs) were downregulated. Cer (d17:1/22:1) was increased. A complete list of differentially expressed metabolites is provided in the Supplementary Table S3. Hierarchical clustering heatmap analysis of differential metabolites (Figure 5G), combined with the differential lipid scatter plot (Figure 5F), indicated a significant shift in metabolic expression patterns before and after treatment, with a predominant trend of lipid downregulation. Variable importance in projection (VIP) analysis, derived from OPLS-DA, was utilized to evaluate the importance of each variable in the model. Based on the VIP bar plot (Figure 5H), the top 5 significant lipid biomarkers identified were CL (20:4/20:3/20:1/20:4), DG (16:2e/14:0), DG (35:0/18:1), TG (18:1/18:2/20:3), and TG (16:0/17:0/18:2). KEGG pathway enrichment analysis, ranked by enrichment ratio, suggested that these differential metabolites may be associated with autophagy, pathogenic *Escherichia coli* infection, Kaposi sarcoma-associated herpesvirus infection, glycosylphosphatidylinositol (GPI) -anchor biosynthesis, cholesterol metabolism, fat digestion and absorption, and regulation of lipolysis in adipocytes (Figure 5I). Receiver Operating Characteristic (ROC) analysis was further employed to assess the diagnostic capacity and accuracy of key lipid biomarkers. The area under the curve (AUC) values closer to 1 indicate a better classification model performance. Differential metabolite analysis identified high-AUC metabolites, including TG (18:2/18:2/18:2) (0.96 [0.8491, 1]), LPI (18:0) (0.96 [0.8491, 1]), MLCL (14:3/20:4/22:6) (0.96 [0.8491, 1]). Detailed ROC analysis results are provided in the Supplementary Table S4.

### 3.6 Western blot

**FIGURE 6:**
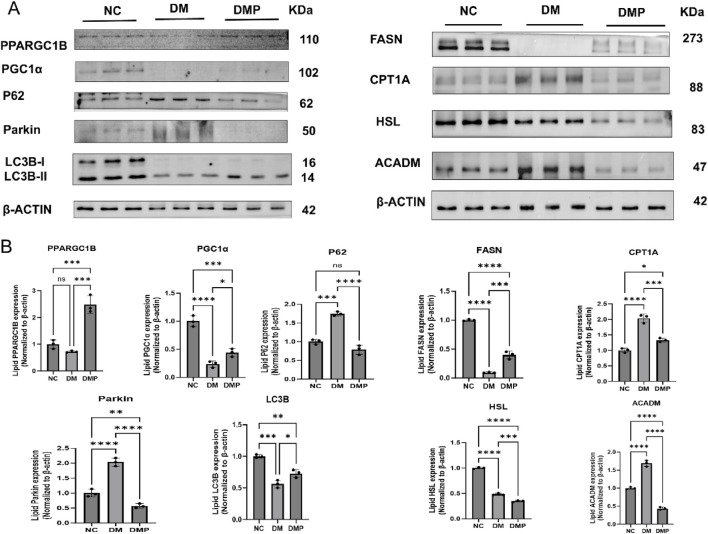
Western blot of FASN,HSL,CPT1A,ACADM, PPARGG1B,LC3B,PGC1α,P62,Parkin protein. **(A)** Western blot of FASN, HSL, CPT1A, ACADM, PPARGG1B, LC3B, PGC1α,P62, Parkin protein level in adipose tissue protein samples. **(B)** Corresponding densitometric analysis in protein. n = 3,*P < 0.05, **P < 0.01, ***P < 0.001, ****P < 0.0001.

To further elucidate the mechanism underlying lipid metabolism regulation, we examined the expression of key proteins associated with lipid metabolism. The results indicated that, in terms of lipid metabolism, FASN and HSL levels were decreased, whereas CPT1A and ACADM levels were increased in the DM group compared to the NC group. In contrast, FASN expression was upregulated in the DMP group compared to the DM group, while HSL, CPT1A, and ACADM levels were downregulated. Furthermore, in the autophagy-related pathways associated with lipid and energy metabolism, P62 and Parkin levels were elevated, while LC3B and PGC1α levels were reduced in the DM group compared to the NC group. However, in the DMP group, PPARGC1B, LC3B, and PGC1α expression levels were increased, whereas P62 and Parkin levels were decreased compared to the DM group (Figure 6).

## 4 Discussion

Diabetes mellitus involves not only impaired glucose metabolism but also pronounced disturbances in lipid metabolism, which are key contributors to the development of insulin resistance, NAFLD, and various diabetic complications. In recent years, PAI-1 has been recognized as a key regulator of metabolic disorders in diabetes, and research on PAI-1 inhibitors as potential therapeutic agents for diabetes is ongoing. Our previous studies demonstrated that PAItrap3, a novel PAI-1 inhibitor, effectively improved blood glucose levels and pancreatic function in diabetic mice (Lin et al., 2025). In this study, we further conducted a systematic analysis of the multidimensional regulatory mechanisms of PAItrap3 on lipid and energy metabolism in diabetic mice.

Metabolic and inflammatory assessments in mice demonstrated that PAItrap3 treatment significantly reduced blood glucose and glycated hemoglobin levels, while increasing insulin levels and markedly decreasing NF-κB, TNF-α, and IL-6 (Lin et al., 2025). Histological analysis further revealed that adipocyte diameter was reduced in the DMP group (HE staining), along with a significant reduction in lipid droplet accumulation (Oil Red O staining), suggesting that PAItrap3 may alleviate adipocyte hypertrophy and inhibit lipid deposition, thereby improving lipid metabolism. Previous studies have also shown that LDL receptor-deficient (ldlr−/−) mice fed a Western diet rich in cholesterol, fat, and sucrose developed obesity, metabolic dysfunction, and atherosclerosis, whereas PAI-1 inhibitor PAI-039 significantly reduced visceral adipose tissue inflammation, hyperglycemia, and hepatic triglyceride content (Khoukaz et al., 2020). Additionally, beyond its role as an energy storage organ, adipose tissue functions as a dynamic and complex endocrine organ, producing and secreting various adipokines (Recinella et al., 2020). During obesity, dysregulation of adipokines leads to a chronic low-grade inflammatory state, which contributes to insulin resistance, diabetes, and its vascular complications (Turpin et al., 2023). Studies have found that hypoadiponectinemia and elevated circulating PAI-1 levels are causally linked to obesity-related insulin resistance and cardiovascular diseases (Li et al., 2011; Vecchiola et al., 2022). In this study, PAI-1 gene expression was downregulated, and inflammatory markers were significantly reduced, further suggesting its potential role in mitigating inflammation. This observation is consistent with our previous studies (Tang et al., 2020).

To further elucidate the metabolic regulatory mechanisms of PAItrap3 in adipose tissue, RNA-Seq analysis was performed, and KEGG pathway enrichment analysis further revealed its role in lipid metabolism remodeling. The solute carrier family 27 member 5 (Slc27a5), also known as long-chain fatty acid transport protein 5 (FATP5), is predominantly expressed in the liver, specifically in the basement membrane of hepatocytes, where it participates in fatty acid transport (Holger et al., 2006). Additionally, fatty acid-binding proteins (FABPs) act as lipid chaperones, mediating lipid uptake, storage, and transport (Maolin et al., 2023). In this study, fatty acid uptake-related genes, including Slc27a5, Fabp1, and Fabp2, were upregulated following PAItrap3 treatment, indicating an enhancement in free fatty acid transmembrane transport and intracellular trafficking, thereby increasing hepatic fatty acid uptake. Angiopoietin-like protein 3 (ANGPTL3) has been identified as a novel therapeutic target for lowering plasma LDL-C and TG (Kersten, 2021). Our findings revealed that lipid metabolism-related genes Acox2, Acot3, Angptl3, and Mttp were upregulated, contributing to enhanced β-oxidation of long-chain fatty acids and promoting hepatic fatty acid degradation. Additionally, hydroxy-methylglutaryl-CoA synthase 2 (Hmgcs2) is a mitochondrial rate-limiting enzyme catalyzing the first step of ketogenesis, and its mild upregulation suggests a potential link to fatty acid β-oxidation (Asif et al., 2022). Regarding cholesterol metabolism, cholesterol acyltransferase/sterol O-acyltransferase 1 (ACAT1/SOAT1) is the key enzyme responsible for cholesteryl ester synthesis in most tissues (Qing et al., 2024). In this study, Soat1 expression was downregulated, suggesting a reduction in cholesteryl ester synthesis, thereby making free cholesterol more readily available for metabolism or conversion into bile acids, which may help regulate hepatic cholesterol balance and prevent excessive hepatic cholesterol accumulation. Bile acids (BAs) play a crucial role in lipid homeostasis, being essential for the absorption and transport of dietary lipids while also regulating metabolic enzymes and transporters critical for lipid modulation, flux, and excretion. BAs have also been identified as regulators of FATP5 (Kumari et al., 2020). Cyp8b1 and Cyp7a1, key cytochrome P450 family proteins, are essential for primary bile acid metabolism (Karen et al., 2019). Their upregulation may facilitate cholesterol conversion into bile acids, thereby reducing cholesterol levels and increasing the bile acid pool. Baat and Acnat2 were upregulated, indicating enhanced bile acid metabolism, while Apoa1 and Mttp upregulation suggests improved lipoprotein assembly and lipid efflux. Collectively, these findings indicate that PAItrap3 treatment remodeled lipid metabolism in adipose tissue, characterized by enhanced fatty acid transport and storage, alongside controlled fatty acid oxidation to maintain energy homeostasis. In future studies, we also plan to investigate other potentially important pathways such as cholesterol metabolism and PPAR signaling.

To further investigate the role of PAItrap3 in regulating lipid metabolism in adipose tissue, we performed qPCR and protein analysis to screen relevant pathways. The results showed that Slc27A5 expression was significantly upregulated in the DMP group compared to the DM group, consistent with RNA-seq findings, while PAI-1 gene expression was downregulated, aligning with the metabolic regulatory effects of PAItrap3 treatment. Protein analysis revealed that PAItrap3 treatment led to decreased expression of HSL (hormone-sensitive lipase) and increased expression of FASN (fatty acid synthase), suggesting inhibited lipolysis and enhanced lipogenesis, which may be attributed to restored insulin levels following PAItrap3 treatment in diabetic mice. Regarding fatty acid oxidation, ACADM (medium-chain acyl-CoA dehydrogenase) plays a role in β-oxidation of fatty acids, while CPT1A (carnitine palmitoyltransferase 1A) is a key enzyme responsible for the transport of fatty acids into mitochondria. After PAItrap3 treatment, CPT1A and ACADM levels decreased, indicating reduced fatty acid oxidation and a metabolic shift toward glucose utilization. Additionally, in fatty acid transport and homeostasis, Slc27A5 (FATP5, fatty acid transport protein 5) is primarily expressed in the liver and adipose tissue, where it facilitates long-chain fatty acid transport and bile acid metabolism (Wu K. et al., 2024). The upregulation of Slc27A5 suggests enhanced fatty acid uptake, which may be linked to increased fatty acid synthesis, as indicated by Acc1 upregulation. Moreover, Perilipin (PLIN1), a lipid droplet-associated protein, protects lipid droplets from degradation by lipases and regulates lipolysis pathways (Yuhui et al., 2024). In this study, Perilipin expression was downregulated, suggesting decreased lipid droplet stability and enhanced lipolysis, possibly leading to increased lipid mobilization and active fatty acid uptake by adipocytes. The decline in Perilipin may represent a compensatory mechanism in energy metabolism, where cells increase fatty acid uptake to sustain energy balance. In diabetic conditions, the effects of PAI-1 inhibition on lipid metabolism appear to be complex, as insulin also plays a significant role in lipid metabolism regulation. In a previous study using obese mice without diabetes, PAI-1 inhibition led to a significant increase in serum glycerol and FFA levels, suggesting enhanced lipolysis. Additionally, HSL and ATGL expression was upregulated, indicating accelerated triglyceride breakdown in adipose tissue (Levine et al., 2021b). Therefore, in non-diabetic obese models, PAI-1 inhibition primarily promotes lipolysis. However, in insulin-deficient diabetic mice, although PAItrap3 treatment partially restored insulin levels, blood glucose remained elevated and did not fully return to normal, indicating persistent metabolic dysregulation. This may explain why PAItrap3 simultaneously improves insulin metabolism and promotes lipogenesis, while also facilitating efficient lipid utilization and reducing excessive fat accumulation, thereby playing a regulatory role in lipid metabolism under diabetic conditions.

Lipid-targeted metabolomics was conducted on white adipose tissue from db/db mice following PAItrap3 treatment, revealing significant lipid metabolic improvements, consistent with the observed lipid profile recovery. KEGG pathway analysis further identified autophagy, fat digestion and absorption, and regulation of lipolysis in adipocytes as key pathways involved in PAItrap3-mediated lipid metabolism regulation. White adipose tissue primarily functions as an energy storage depot, characterized by low mitochondrial density, with its metabolic function closely linked to fatty acid oxidation and glucose metabolism. Autophagy plays a crucial role in energy and lipid metabolism, as studies have demonstrated that lipid droplets and autophagic components interact during nutrient deprivation, and inhibition of autophagy in cultured hepatocytes and mouse liver increases triglyceride accumulation in lipid droplets (Singh et al., 2009). Moreover, PAI-1 knockout reduced the number of M1-type macrophages, increased M2-type macrophages, and was associated with macrophage autophagy (Fang et al., 2024). To further investigate the key autophagy and energy metabolism pathways, we screened relevant markers and found that mitophagy and energy metabolism-related proteins (PGC1α and PPARGC1B) were upregulated, while autophagy activity was enhanced (LC3B ↑, p62 ↓), suggesting that PAItrap3 may promote autophagy to clear metabolic waste and improve lipid homeostasis. PGC1α and PPARGC1B/PGC1β, as coactivators of peroxisome proliferator-activated receptors (PPARs), regulate mitochondrial function and energy metabolism and are essential in energy expenditure (EE), fatty acid oxidation (FAO), and metabolic switching between lipid and glucose utilization (Arany et al., 2005). LC3B (microtubule-associated protein 1 light chain 3B) and p62 (SQSTM1, Sequestosome 1) are classical markers of autophagy activity, where increased LC3B indicates enhanced autophagosome formation, and decreased p62 suggests active substrate degradation, leading to lipid droplet autophagy (lipidophagy). A previous study found that canagliflozin induced lipidophagy by increasing LC3-II levels while reducing p62 and perilipin 2 levels in rats and HepG2 cells, alongside suppression of mTOR expression in the rat liver (El-Ashmawy et al., 2025). Autophagy directly degrades lipid droplets, releasing free fatty acids for mitochondrial β-oxidation, while PGC1α enhances mitochondrial oxidative capacity, preventing lipid toxicity (lipotoxicity) from excessive intracellular free fatty acid accumulation. Additionally, Parkin, an essential ubiquitin ligase regulating mitophagy, tags damaged mitochondria for autophagic degradation, thereby maintaining mitochondrial quality (Liu et al., 2025). In this study, Parkin levels were reduced, potentially indicating its depletion due to enhanced mitophagy. Collectively, these findings suggest that PAItrap3-mediated autophagy-lipid metabolism regulation may explain the observed increase in β-oxidation of fatty acids. While PAItrap3 enhances glucose utilization, it also modulates lipid metabolism in db/db diabetes mice through activation of the PGC1α/PPARGC1B-autophagy pathway, further supporting its potential role in metabolic regulation.

Changes in lipid metabolites represent the final metabolic response of the body to external interventions and are also key metabolic indicators of disease progression in T2DM (Zhong et al., 2024). Additionally, disruptions in lipid metabolism are closely associated with diabetic complications, as studies have shown that Cer (d18:0/22:0) and Cer (d18:0/24:0) are independent risk factors for the occurrence of diabetic retinopathy in T2DM patients (He et al., 2024). In this study, targeted lipidomics revealed that following PAItrap3 treatment, most triglycerides (TGs) were downregulated, while TG (15:0/16:1/16:1), TG (15:0/18:2/22:6), TG (18:1/10:3/18:2), and TG (16:0/18:2/18:2) were upregulated. Additionally, among diacylglycerols (DGs), only DG (34:0/18:3) was upregulated, supporting the role of PAItrap3 in improving lipid metabolism. Notably, the upregulated lipid species were primarily enriched in polyunsaturated fatty acids (PUFAs), which play crucial roles in immune regulation (Kimsor et al., 2024), anti-inflammatory processes (Calder, 2013), membrane stability, and energy metabolism modulation (Harwood, 2023), serving as energy buffers and antioxidant reserves. Meanwhile, most cardiolipins (CLs) were downregulated, except for CL (20:4/20:3/20:1/20:4). CL is a hallmark lipid of the mitochondrial inner membrane, directly influencing mitochondrial function and respiratory chain complex activity. In a choline-deficient diet (CDD)-induced fatty liver model, CL content was decreased in liver mitochondria, whereas oxidized CL levels were elevated. Additionally, studies found that the reduced activity of complex I in liver mitochondria from CDD-fed animals could be restored to control levels by exogenous CL supplementation (Petrosillo et al., 2007), suggesting that the upregulation of CL may serve as a compensatory response to oxidative stress, helping to maintain mitochondrial membrane stability. In diabetic mouse models, the widespread reduction in CL may be attributed to severe energy metabolism disturbances and mitochondrial damage, while the activation of autophagy and energy metabolism pathways may have contributed to increased lipid consumption, aligning with the observed decline in Parkin expression. As diabetes and energy metabolism reach a more stable state, autophagy and lipid metabolism may undergo further remodeling. The top three metabolites with the highest AUC values were TG (18:2/18:2/18:2), LPI (18:0), and MLCL (14:3/20:4/22:6), all of which were downregulated, suggesting their potential role as key biomarkers of PAItrap3-mediated metabolic remodeling. Among these, TG (18:2/18:2/18:2) is enriched in linoleic acid (LA, 18:2, n-6). Studies have shown that excessive intake of LA can lead to the production of oxidized linoleic acid metabolites (OXLAMs), impair mitochondrial function, and potentially contribute to the development of various chronic diseases (Mercola and D'Adamo, 2023). Therefore, the reduction in LA levels may be closely related to the improvement of inflammation. Additionally, MLCL (monolysocardiolipin) is a key intermediate in mitochondrial cardiolipin (CL) remodeling, and given that most CL species were downregulated, MLCL, as a metabolic intermediate of CL, was also reduced accordingly. On the other hand, studies have found that obesity, insulin resistance, and elevated plasma levels of LPI (lysophosphatidylinositol) are closely linked (Son et al., 2024). In visceral adipose tissue, LPI induces the expression of genes associated with fat deposition, promoting lipid accumulation, while it is also implicated in excessive insulin secretion (Arifin and Falasca, 2016). Therefore, the decrease in these lipid metabolites may not only result from improved glycemic control and increased fatty acid oxidation but could also be associated with enhanced insulin sensitivity, reduced inflammation, and an increased need for mitochondrial cardiolipin remodeling.

However, this study has certain limitations. First, as the research was primarily conducted using db/db mouse models, its applicability to human metabolic diseases requires further validation. Second, the long-term safety and tissue-specific effects of PAItrap3 remain to be assessed, particularly regarding its differential effects across various tissues such as the liver, muscle, and adipose tissue. Future studies should incorporate functional experiments, such as gene knockout models, to further elucidate the precise role of PAItrap3 in lipid metabolism regulation, thereby refining its potential clinical applications for diabetes treatment.

## 5 Conclusion

**FIGURE 7:**
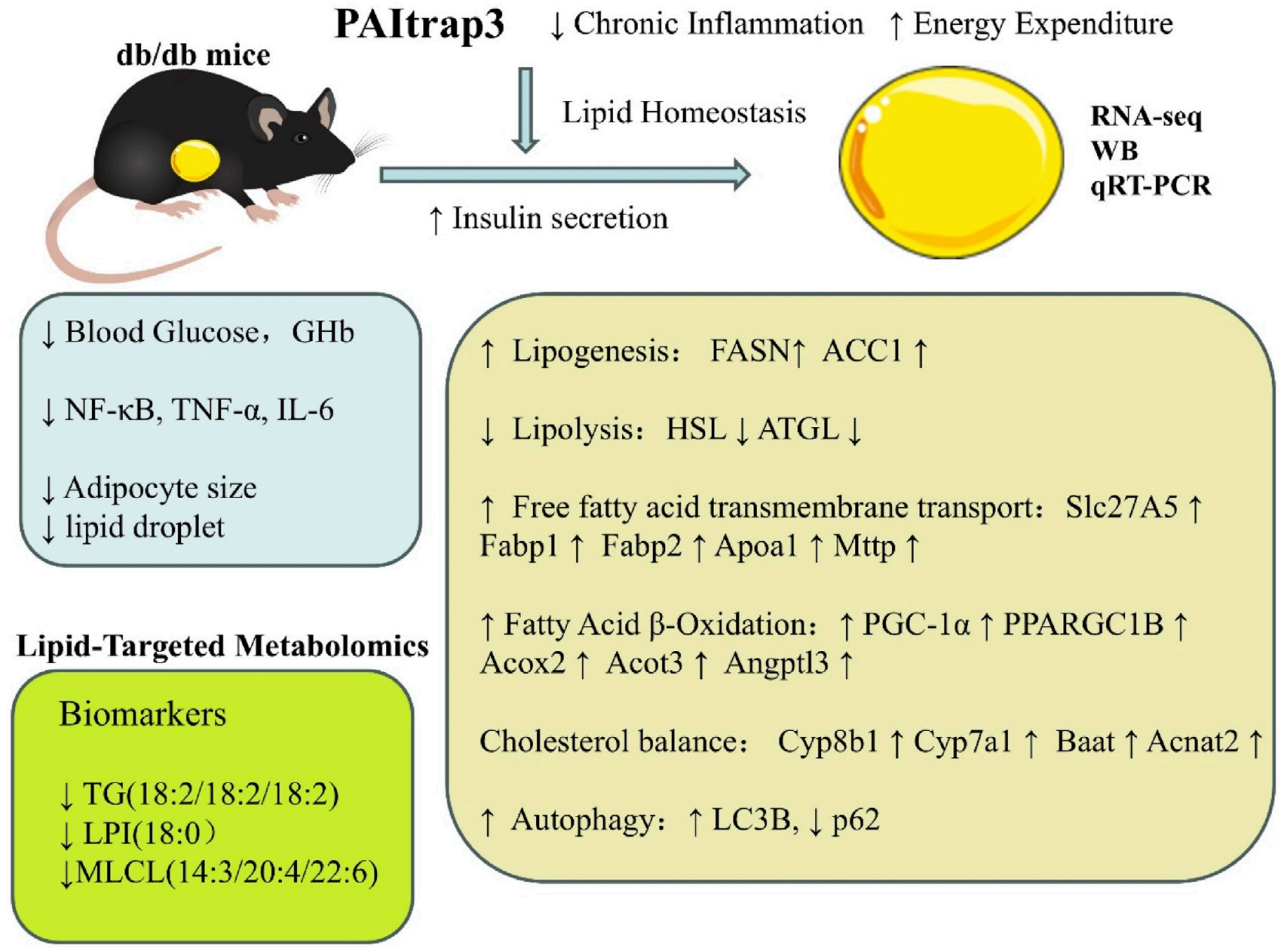
The Mechanism of PAItrap3 in Lipid Metabolism of db/db Mice.

This study demonstrates that PAItrap3, a PAI-1 inhibitor, improves metabolic dysfunction in diabetic mice by modulating lipid metabolism and autophagy. Following PAItrap3 treatment, blood glucose levels were significantly reduced, insulin levels improved, inflammatory markers decreased, and lipid droplet accumulation in adipose tissue was reduced. Mechanistically, PAItrap3 may alleviate chronic inflammation and enhance insulin sensitivity, while simultaneously promoting fat storage and restoring glucose metabolism. Additionally, it activates autophagy and mitochondrial function, facilitates fatty acid β-oxidation, and optimizes energy metabolism (Figure 7).

## Data Availability

The lipidomic data presented in the study are deposited in the Figshare repository, doi: 10.6084/m9.figshare.29205749. The RNA-seq data presented in the study are deposited in the Figshare repository, doi: 10.6084/m9.figshare.29205809.
